# Ebselen: A Promising Repurposing Drug to Treat Infections Caused by Multidrug-Resistant Microorganisms

**DOI:** 10.1155/2024/9109041

**Published:** 2024-03-30

**Authors:** Agostinho Alves de Lima e Silva, André Rio-Tinto

**Affiliations:** ^1^Laboratory of Biology and Physiology of Microorganisms, Biomedical Institute, DMP, Federal University of the State of Rio de Janeiro (UNIRIO), Rio de Janeiro 20211-030, Brazil; ^2^Laboratory of Pathogenic Cocci and Microbiota, Instituto de Microbiologia Paulo de Góes, Universidade Federal do Rio de Janeiro (UFRJ), Rio de Janeiro 21941-853, Brazil

## Abstract

Bacterial multiresistance to drugs is a rapidly growing global phenomenon. New resistance mechanisms have been described in different bacterial pathogens, threatening the effective treatment of even common infectious diseases. The problem worsens in infections associated with biofilms because, in addition to the pathogen's multiresistance, the biofilm provides a barrier that prevents antimicrobial access. Several “non-antibiotic” drugs have antimicrobial activity, even though it is not their primary therapeutic purpose. However, due to the urgent need to develop effective antimicrobials to treat diseases caused by multidrug-resistant pathogens, there has been an increase in research into “non-antibiotic” drugs to offer an alternative therapy through the so-called drug repositioning or repurposing. The prospect of new uses for existing drugs has the advantage of reducing the time and effort required to develop new compounds. Moreover, many drugs are already well characterized regarding toxicity and pharmacokinetic/pharmacodynamic properties. Ebselen has shown promise for use as a repurposing drug for antimicrobial purposes. It is a synthetic organoselenium with anti-inflammatory, antioxidant, and cytoprotective activity. A very attractive factor for using ebselen is that, in addition to potent antimicrobial activity, its minimum inhibitory concentration is very low for microbial pathogens.

## 1. Introduction

In recent decades, there has been an alarming increase in the number of nosocomial infections and morbidity and lethality caused by multidrug-resistant pathogens, with a major impact on health worldwide and worrying projections for the next 30 years. The vertiginous and global growth in the emergence of multidrug-resistant (MDR) microorganisms, characterized by multiple resistance to different classes of drugs, is one of the greatest current challenges in public health [1].

In bacteria, resistance to antimicrobials can be acquired through mutations in genes or the acquisition of exogenous DNA. Regarding mutations, resistance acquisition is more limited. Meanwhile, the acquisition of exogenous DNA has a much greater impact because it involves the transfer of chromosomal genes or mobile transferable genetic elements, such as R plasmids, which can spread intra- and inter-species. In both mechanisms, the problem is greatly amplified by the misuse or abuse of antimicrobials, which promotes the selection of resistant clones.

Drug resistance reports in fungi have also become increasingly common. Although less studied, the evolutionary process of resistance in these microorganisms is also governed mainly by an adaptive response to the selective pressure of using antifungals, and it is considered that all pathogenic fungi can acquire resistance [2]. However, unlike bacteria, there is no horizontal gene transfer between these microorganisms, which makes resistance development relatively slower, combined with more limited transmission between patients [3], except *Candida auris* [4].

According to the World Health Organization (WHO) [5], the bacteria with MDR profile comprising the list of priority pathogens for the development of new antibiotics include (a) priority 1 (critical): carbapenemase-resistant *Acinetobacter baumannii* and *Pseudomonas aeruginosa* and Enterobacteriaceae (carbapenemase-resistant and strains that produce extended-spectrum beta-lactamases (ESBLs)), and (b) priority 2 (high): vancomycin-resistant *Enterococcus faecium*; methicillin-resistant *Staphylococcus aureus* (MRSA) and strains with intermediate or total vancomycin resistance; clarithromycin-resistant *Helicobacter pylori*; fluoroquinolone-resistant *Campylobacter* spp.; fluoroquinolone-resistant *Salmonellae*; and cephalosporin-resistant and fluoroquinolone-resistant *Neisseria gonorrhoeae*.

The rapid evolution of drug resistance and the borderless spread of multiresistant pathogens require an urgent improvement in the effectiveness of current treatment protocols and the implementation of new therapeutic strategies to overcome the shortcomings of current schemes. One promising approach to tackling this problem is the so-called repositioning or repurposing of drugs, defined as investigating new uses for existing drugs [6].

Studies have shown that many drugs used for different primary purposes in medical practice have antimicrobial activity [7]. Thus, repositioning for such drugs is advantageous since their pharmacokinetic/pharmacodynamic and toxicity characteristics are already well characterized. Furthermore, we must consider that the time required to develop new antimicrobials is considerably longer, the costs involved are high, and despite this, the results obtained are often unsatisfactory.

**Figure 1:**
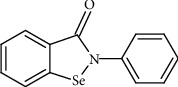
Chemical structure of ebselen.

Examples of drugs that can show a greater or lesser degree of activity against microbial pathogens include drugs belonging to the nonsteroidal anti-inflammatory class [8, 9], antihistamines [10], antineoplastics and antipsychotics/antidepressants [6], anesthetics and statins [7], organometallic compounds, such as auranofin (a drug approved for the treatment of rheumatoid arthritis), and organoselenium compounds, which have different pharmacological activities [11]. Among the selenorganic compounds already synthesized, ebselen (2-phenyl-1,2-benzisoselenazol-3(2H)-one) (Figure 1) has been the most studied, mainly due to its antioxidant properties, but also anti-inflammatory and anti-atherosclerotic properties [12].

**Figure 2:**
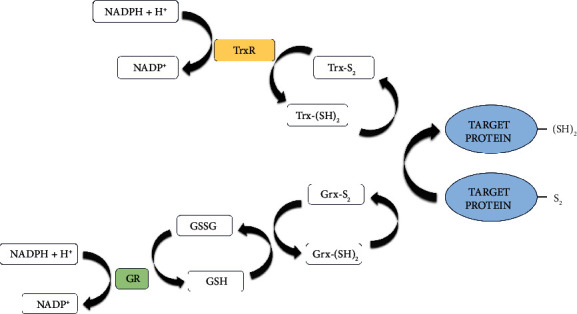
General components of the thioredoxin system (top) and glutaredoxin system (bottom). TrxR: thioredoxin reductase; Trx: thioredoxin; GR: glutathione reductase; GSH: glutathione; GSSG: oxidized GSH; Grx: glutaredoxin. TrxR catalyzes the electron transfer from NADPH to Trx, which then reduces downstream targets. GR catalyzes the reduction of GSSG to GSH, which can be used by GSH-dependent enzymes, such as Grx. In prokaryotes, ebselen acts as a TrxR inhibitor, leading to the accumulation of reactive oxygen species. GSH-negative bacteria, such as Gram-positive, are most affected by ebselen.

The antibacterial mechanism of action of ebselen involves the thiol-dependent thioredoxin (Trx) system [11], which comprises the proteins thioredoxin (Trx) and thioredoxin reductase (TrxR) and NADPH as an electron donor. This antioxidant system plays an important reductive role in proteins involved in the oxidative stress control process, DNA synthesis, and protein repair. In addition to Trx, the cellular redox environment is also controlled by the thiol-dependent glutathione/glutathione reductase (GSH/GR) system [13] (Figure 2), and the mammalian Trx and GSH systems can cross-supply electrons and serve as a backup system for each other [14]. The Trx reduction is mediated by the enzyme thioredoxin reductase (TrxR), which differs substantially between higher eukaryotes and microorganisms. On the other hand, the GSH/GR system is absent in many bacteria, especially most Gram-positive bacteria, which makes TrxR essential for their survival under oxidative stress conditions [11, 14].

Ebselen acts competitively by inhibiting bacterial TrxR [11, 15]. Thus, its effect is more successful on microorganisms lacking GSH/GR. This drug has also shown action by inhibiting protein synthesis. In *S. aureus*, it also showed secondary effects on nucleic acids, lipid synthesis, and, to a lesser extent, cell wall synthesis [16]. In addition to antibacterial activity, ebselen also has antifungal and antiviral action [17]. Several clinical trials have analyzed ebselen for the treatment of different diseases, and so far, this drug has been shown to be safe at recommended doses [17–25].

## 2. Ebselen Antifungal Activity

In addition to bacterial pathogens, ebselen's potent antimicrobial action has been described for fungal infectious agents. Different mechanisms of antifungal action have been proposed for ebselen. These include inhibition of the plasma membrane H^+^-ATPase pump (Pma1p) in yeast [26, 27], activation of DNA damage response and alteration in nuclear proteins [28], induction of ROS through inhibition of glutamate dehydrogenase [29], and inhibitory activity through depletion of intracellular GSH, leading to increased production of reactive oxygen species (ROS), thus disrupting redox homeostasis [30].

According to a systematic review by Benelli [31], a wide spectrum of fungi have shown in vitro susceptibility to ebselen, including *Candida* spp., *Cryptococcus* ssp., *Trichosporon* spp., *Aspergillus* spp., *Fusarium* spp., *Pythium* spp., and *Sporothrix* spp. This review described the antifungal activity of ebselen at concentrations ≤64 *μ*g/mL against 96% of the pathogenic fungi evaluated in different studies. The geometric mean of the minimum inhibitory concentration (MIC) for yeasts showed lower values (0.29 to 3.47 *μ*g/mL) than for filamentous fungi (4.87 to 11.59 *μ*g/mL).

An older study reported a relatively high MIC of ebselen for *C. albicans* (8 *μ*g/ml) [32] compared to later studies. For example, Thangamani et al. [30] demonstrated that this compound showed potent antifungal activity against clinically relevant isolates of *Candida* and *Cryptococcus* at concentrations ranging from 0.5 to 2 *μ*g/ml. In two models of infection with *Caenorhabditis elegans*, ebselen showed superiority in reducing the fungal load compared to the conventional antifungals such as fluconazole, flucytosine, and amphotericin. In another study, to overcome the drug's low aqueous solubility, Jaromin et al. [33] used an ebselen delivery system through encapsulation in nanocapsules for topical use. The ebselen nanocapsules were active against strains of *C. tropicalis*, *C. albicans*, and *C. parapsilosis*, with MIC values around 4.2 and 1.25 times lower than free ebselen, respectively. The authors suggested this procedure as a promising, safe, and complementary alternative to treating cutaneous candidiasis.

In a screening from the Prestwick Chemical Library, a reuse library of 1280 small molecules comprising mainly off-patent approved drugs, ebselen was identified as a drug with potent antifungal activity. It showed 100% growth inhibition of *C. auris*, an emerging global multidrug-resistant pathogen, at concentrations as low as 2.5 *μ*M [34]. Furthermore, ebselen also showed activity in this study against *C. dubliniensis*, *C. parapsilosis*, *C. tropicalis*, *C. glabrata*, *C. lusitaniae*, and *C. krusei*, with 50% inhibitory concentration (IC50) in the range from 0.5 to 2 *μ*g/ml. Another study concerning the effect of ebselen on *C. auris* showed MIC90% of 4 *μ*g/ml for six strains and 8 *μ*g/ml for one strain [35]. On the other hand, ebselen was also very active against strains of the fungus *Trichosporon asahii*, with MIC ranging from 0.25 to 8.0 *μ*g/mL [36]. Meanwhile, MIC ranged from 0.06 to 4 µg/mL for isolates of echinocandin-resistant *C. parapsilosis* [37].

Regarding filamentous fungi, we point out reports of the inhibitory activity of ebselen against *A. niger* (17 *μ*g/mL) [32]; *Fusarium* spp. (2–8 *μ*g/ml) [38]; *A. flavus*, *A nidulans*, and *A. terreus* (6.25, 1.56, and 156 *μ*g/mL, respectively) [39]; *A. fumigatus*, *Fusarium* spp., *Scedosporium apiospermum*, and *Rhizopus arrhizus* (4 *μ*g/ml, 4–8 *μ*g/ml, 8 *μ*g/ml, and 16 *μ*g/ml, respectively) [34]; *A. fumigatus* (16–64 *μ*g/ml) [40]; and *Trichophyton mentagrophytes* (MIC geometric means of 0.442 *μ*g/mL and 0.518 *μ*g/mL for the human and animal strains, respectively) [41]. Furthermore, it is worth noting that an in vivo study showed that ebselen protected against invasive aspergillosis in a murine model, with efficacy and safety similar to conventional antifungal drugs [42].

## 3. Ebselen Activity on Bacteria

Nozawa et al. [43] suggested that the presence of selenium in the ebselen molecule is essential for its antibacterial action since a corresponding sulfur analogue of this compound (PZ 25) lost its antioxidant activity and showed a weak antibacterial effect compared to ebselen.

As mentioned above, there are two important thiol-dependent enzyme systems involved in redox control in eukaryotic and prokaryotic cells: Trx and GSH. These systems transfer electrons from NADPH to their substrates through TrxR and GR and are significantly different between prokaryotes and higher eukaryotes. For example, bacterial TrxR contains cysteine in its active site. Meanwhile, mammalian TrxR contains a rare amino acid (selenocysteine) in its active site [44]. Moreover, bacterial TrxR (and from helminths, fungi, and some protozoa) has a low molecular weight, unlike the high molecular weight enzyme found in mammals [11]. On the other hand, in addition to Trx, mammalian cells also contain the GSH antioxidant system, which is absent in Gram-positive bacteria. Ebselen acts as a substrate for mammalian TrxR. However, it is a potent inhibitor of the bacterial TrxR enzyme, with selective antibacterial action, especially for those that are GSH-negative, such as Gram-positive bacteria [15].

In Gram-negative bacteria, except for *H. pylori*, the Trx system is supplemented by the GSH system, which is also capable of neutralizing reactive oxygen and nitrogen species. Consequently, while ebselen exerts bactericidal effects in Gram-positive bacteria through the inhibition of TrxR, resulting in the accumulation of ROS, Gram-negative bacteria rely on the GSH system as a supplementary mechanism to counteract TrxR inhibition and regulate the redox balance. This feature renders Gram-negative bacteria less susceptible to the detrimental effects of oxidative stress compared to Gram-positive bacteria [45].

The significance of TrxR and GSH in influencing susceptibility to ebselen was demonstrated by Lu et al. [15] in their study on *E. coli*, a bacterium found to be approximately 100 times more resistant to ebselen than *S. aureus*. Experiments conducted with *E. coli* DHB4 mutants with disrupted GSH production revealed that the *gor*^−^ strain (lacking GR) and the gshA null mutant (*gshA*^−^, deficient in GSH) exhibited heightened susceptibility to ebselen compared to the wild-type strain. Conversely, mutants based only on TrxR for electron flow to ribonucleotide reductase were notably more sensitive to growth inhibition by ebselen, whereas mutants based on GSH were considerably less sensitive. Furthermore, it has been suggested that the outer membrane of Gram-negative bacteria could act as a barrier that reduces the ability of TrxR-inhibiting drugs, such as ebselen and auranofin [46, 47], to penetrate the cell.

The hydrophobic nature of the outer membrane may contribute to its increased impermeability to drugs such as ebselen and auranofin. Chen and Yang [46] conducted a study where they synthesized several ebselen analogues with varying polarities and evaluated their efficacy against Gram-negative pathogens. Some analogues with higher hydrophilicity exhibited remarkable antibacterial activity against multidrug-resistant Gram-negative bacteria, notably *E. coli*-ESBL and carbapenem-resistant strains of *E. coli* producing NDM-1, surpassing the effectiveness of conventional antibiotics. Furthermore, through time-kill kinetics studies, accumulation assays, and scanning electron microscopy imaging, it was observed that the 4i analogue could penetrate bacterial membranes, inducing irregular cell morphology and rapid demise of *E. coli*-ESBL and *E. coli*-NDM-1. Hence, ebselen analogues possessing polarity represent a promising approach to combat infections caused by multidrug-resistant Gram-negative pathogens.

Conversely, in Gram-positive bacteria, the inhibition of protein synthesis appears to play a significant role in the antibacterial activity of ebselen. Thangamani et al. [16] demonstrated, via a macromolecular synthesis assay, that ebselen inhibited protein synthesis in *S. aureus* at concentrations equivalent to the MIC. Moreover, at higher concentrations (8x MIC), secondary effects on DNA, RNA, lipid synthesis, and, to a lesser extent, cell wall synthesis were also observed. According to the authors, it is possible that disruption of protein synthesis could lead to downstream inhibition of other pathways. Although the exact mechanism of action of ebselen in *S. aureus* remains unclear, it is noteworthy that besides targeting Trx-R, this organoselenium compound can react with various protein thiols, forming selenosulfide bonds, as well as with thiols of low molecular mass [48].

*Mycobacterium tuberculosis* is another pathogen whose genome does not encode components of the GSH system [49]. It is interesting to notice that the main inhibition target of ebselen for this microorganism does not seem to be TrxR, but proteins of the antigen 85 complex (Ag85), which are involved in the synthesis of two important components of the wall of *M. tuberculosis*, mycolylarabinogalactan, and trehalose dimycolate [50]. Moreover, the transpeptidase LdtMt2 from *M. tuberculosis* has been identified as a potential inhibitory target for ebselen [51].

### 3.1. Susceptibility of Gram-Positive Bacteria and Mycobacteria to Ebselen

Nozawa et al. [43] provided the first evidence of the susceptibility of Gram-positive bacteria to ebselen, with MICs for clinical isolates of *Streptococcus agalactiae*, *Streptococcus salivarius*, *Streptococcus pneumoniae*, and *E. faecium* ranging from 1.56 to 6.25 *μ*g/mL. Meanwhile, for *S. aureus* and *S. epidermidis*, the MIC was 0.20 *μ*g/mL. Even though most studies on the MIC of ebselen use the broth microdilution technique, these results were obtained using the plate dilution method.

Then, ebselen's bactericidal activity has also been reported in clinical isolates of multidrug-resistant *S. aureus*, including MRSA and VRSA (vancomycin-resistant *S. aureus*) strains, in addition to strains resistant to linezolid or mupirocin. The MICs ranged from 0.125 *μ*g/ml to 0.5 *μ*g/ml [16]. The MICs ≤1 *μ*g/ml (mostly between 0.125–0.5 *μ*g/mL) were also detected for clinical isolates of *Staphylococcus*, *Streptococcus*, and *Enterococcus* [52]. Similarly, in a comprehensive screening of a library containing 727 FDA-approved drugs and small molecules, the MIC90 of ebselen was only 0.25 *μ*g/mL for multidrug-resistant *S. aureus* clinical isolates [53]. The MICs ranging from 0.25 to 1.0 *μ*g/mL were also detected for clinical isolates of *S. aureus*, with minimum bactericidal concentrations of only one dilution of their MICs for all tested isolates [54]. Dong et al. [55] observed a slightly higher MIC of ebselen (2.2 *μ*g/mL) for a clinical isolate of *S. aureus*. In contrast, Chen and Yang [46] found, for the compound ebselen 4a that they synthesized, an MIC of 64 *μ*g/mL for an MRSA isolate and a VRE. Meanwhile, the MIC for a clinical isolate of *S. aureus* was only 0.5 *μ*g/mL.

In addition to strong antibacterial activity toward *S. aureus*, *Bacillus cereus*, and *M. tuberculosis*, interestingly, prolonged exposure of *S. aureus* and *B. cereus* to ebselen did not induce resistance [56]. According to the authors, the high barrier against the development of resistance to ebselen indicates that there are very few substitutions allowed in the target or that several targets are affected simultaneously. Corroborating this finding, Mohammad et al. [57] showed that against two different strains of *S. aureus*, resistance to ebselen did not emerge after 14 consecutive passages, unlike resistance to mupirocin, which emerged after only five passages. Moreover, serial passage experiments conducted with ebselen over a period of 14 days using a clinical isolate of vancomycin-resistant *E. faecium* did not lead to the development of resistance to the organoselenium compound [58]. One possible explanation for this is the essential nature of the TrxR target for ebselen in Gram-positive bacteria. Furthermore, a simultaneous impact on other cellular targets may be considered, preventing the emergence of resistance to organoselenium. Currently, there is a lack of data in the literature regarding microorganisms that have acquired resistance following short or prolonged exposure to ebselen. Nonetheless, it is imperative to acknowledge that only a limited number of studies addressing this matter have been conducted thus far.

Even though the MIC values of ebselen described for *M. tuberculosis* can potentially be considered within a therapeutic concentration range, overall, they are higher than those described for Gram-positive bacteria [56]. Lu et al. [15] detected MICs of 10 and 20 *μ*g/ml for this pathogen. Meanwhile, Zhu et al. [59] reported MICs of 50 *μ*M (13.66 *μ*g/ml) for two clinical strains of *M. tuberculosis* (MDR—multidrug-resistant and XDR—extensively drug-resistant) and 100 *μ*M (27.32 *μ*g/ml) for *M. tuberculosis* H37Rv.  Table 1 shows some characteristics of the tested strains and MICs relating to the antimicrobial activity of ebselen on Gram-positive pathogens, such as *Staphylococcus*, *Enterococcus*, and *Clostridium*, as well as on *M. tuberculosis*.

Ebselen has also shown antimicrobial activity in studies using in vivo models. In orally treated mice, this organoselenium significantly increased survival in a lethal model of septicemic MRSA infection [53]. It was able to substantially reduce the bacterial load of VRE in fecal samples after just three days of treatment [58].

A significant reduction in the infectious load of MRSA (85%) was also observed in an invertebrate study model of *Caenorhabditis elegans* [52]. In mice subjected to systemic intoxication with a lethal dose of the TcdB toxin from *Clostridioides* (*Clostridium*) *difficile*, ebselen protected the animals. Meanwhile, the untreated group showed 60% mortality in the first 24 hours and 100% mortality at the end of day 2 [60]. Ebselen not only inhibited the toxins of *C. difficile* but determined the death of pathogenic *C. difficile* by disrupting its redox homeostasis and altering the normal concentrations of NAD^+^ and NADH, which are essential for many metabolic functions in cells [61]. Nevertheless, these authors cautioned that blood present in the anaerobic culture medium, as recommended for sensitivity tests with this microorganism, might obscure the activity of ebselen. Additionally, they suggested that the diminished activity of ebselen in blood could potentially compromise its antibacterial efficacy in vivo.

In rodents, applying ebselen to uncomplicated skin wounds significantly reduced the MRSA load and the levels of proinflammatory cytokines [16, 55]. On the other hand, in two models of obese and diabetic mice, topical treatment with ebselen on MRSA-infected pressure ulcers showed similar results to oral linezolid in obese animals but was less effective in diabetics. In both animals, the results were inferior to those of topical mupirocin, and the authors suggested that a higher dose of ebselen, or longer treatment, may be required to obtain an effect similar to that of mupirocin [57].  Table 2 shows some data from in vivo studies with ebselen.

### 3.2. Ebselen's Effect on Gram-Negative Pathogens

Unlike Gram-positive bacteria, Gram-negative bacteria are less susceptible to organoselenium compounds. In one of the first reports on the antibacterial activity of ebselen for Gram-negative bacteria, the MIC of Gram-positive bacteria, such as *S. aureus* and *S. epidermidis*, was in the order of nanograms. Meanwhile, for members of the Enterobacteriaceae family, it ranged from 12.5 to 50 *μ*g/mL [43]. However, *H. pylori* is an exception among the Gram-negative bacteria, possibly due to the lack of the GSH system in this species [14]. For example, ebselen inhibited a clinical strain of *H. pylori* with 3.13 *μ*g/ml, and its sulfur analogue, ebsulfur, showed an MIC value as low as 0.39 *μ*g/ml [15].

Considering other Gram-negative bacteria, compared to what has been reviewed for Gram-positive bacteria, the MIC of ebselen is relatively or quite high (Table 3). For example, Piętka-Ottlik et al. [32] reported an MIC value of 274 *μ*g/ml for *E. coli*. In a study in progress, we found MICs of ≥50 *μ*g/ml for clinical strains of *Acinetobacter baumanii* (unpublished data).

Despite the lower susceptibility of Gram-negative bacteria to ebselen, it is worth noting the strong synergistic antibacterial effect when this organoselenium is associated with silver [62–65]. It is also important to consider that the presence of ebselen can drastically decrease the silver concentration required for antibacterial activity, with highly significant selective toxicity in bacteria on mammalian cells, which should facilitate the systemic medical application of silver in the treatment of infections by Gram-negative MDR bacteria [65].

The antibacterial properties of silver ions (Ag^+^) have been recognized for centuries, with historical examples such as the ancient Greeks employing silver for wound treatment. Experimental investigations have revealed various microbial targets for silver activity, encompassing DNA, proteins, and small molecules [68]. In the past decade, Morones-Ramirez et al. [69] demonstrated that silver disrupts numerous bacterial cellular processes, including disulfide bond formation, metabolism, and iron homeostasis, leading to oxidative stress, heightened production of reactive oxygen species (ROS), and enhanced membrane permeability in Gram-negative bacteria. Additionally, Ag^+^ has been shown to potentiate the efficacy of conventional bactericidal antibiotics both in vitro and in vivo, effectively restoring antibiotic susceptibility in resistant bacterial strains.

More recent research involving five Gram-negative pathogens, each possessing GSH, unveiled the synergistic bactericidal effects of combining ebselen with Ag^+^, which directly interfered with both the thioredoxin (Trx) system and the GSH system. For instance, the combination of 5 *μ*M Ag^+^ and 20 *μ*M ebselen resulted in loss of TrxR and Trx activities, along with depletion of functional GSH within 10 minutes compared to the control, surpassing the individual activities of ebselen or Ag^+^. This combination also elicited substantially elevated ROS levels. Furthermore, the efficacy of this combination was demonstrated in a murine model of peritonitis caused by *E. coli* [65].

Conversely, contrasting findings were observed regarding the combination of silver with conventional antibiotics in Gram-negative pathogens, wherein the synergistic antibacterial effects were linked to silver's blockade of the bacterial Trx system, with no discernible direct effects on the GSH system. While the combination of antibiotics with silver augmented antibacterial effects by enhancing ROS production, its efficacy was inferior to that observed for the ebselen-silver combination. The compromise of both the Trx and GSH systems induced by the combination of organoselenium with the metal may account for this outcome.

Dong et al. [63] demonstrated that the synergistic combination of ebselen and silver induced GSH depletion and TrxR inhibition in a strain of *Yersinia pseudotuberculosis*, leading to deformation, shrinkage, and cytoplasmic leakage, indicative of cell membrane rupture. Additionally, this combination significantly reduced bacterial burdens in a mouse model of gastroenteritis caused by the bacteria [63]. Synergistic bactericidal combinations, characterized by GSH depletion and TrxR inhibition in Gram-negative bacteria, have been also reported for *Acinetobacter baumannii* [62] and uropathogenic *Escherichia coli* [64]. Additionally, akin to silver ions, silver nanoparticles exhibit robust inhibitory effects on bacterial thioredoxin reductase and demonstrate synergistic interactions when combined with ebselen [70, 71].

As mentioned above, another pathway for the potential use of organoseleniums against Gram-negative bacteria involves using hydrophilic derivatives of ebselen. Polar analogues of this compound showed strong antibacterial activity against multidrug-resistant Gram-negative bacteria, particularly ESBL-producing strains of *E. coli* (MIC = 1–4 *μ*g/mL) and *E. coli* NDM-1 (MIC = 4–32 *μ*g/mL), being more potent than traditional antibiotics such as cefazolin and imipenem [46].  Table 2 shows data from in vivo studies of ebselen with Gram-negative bacteria.

## 4. Ebselen and Classical Antibiotics

Ebselen primarily targets Gram-positive bacteria, making it most relevant to compare its potency and toxicity with conventional antibiotics within this bacterial group. For instance, Thangamani [52] demonstrated that the efficacy of ebselen against *Staphylococcus* and *Enterococcus* surpassed that of vancomycin and linezolid, two primary drugs for treating these pathogens, with MIC90 values in the nanogram range (0.25–0.5 *μ*g/mL), irrespective of the strains' antibiotic resistance phenotype. Comparing this activity of ebselen with the CLSI guidelines for the two antibiotics [72], the susceptibility cutoff points for vancomycin and linezolid in *S. aureus* are ≤2 *μ*g/mL and ≤4 *μ*g/mL, respectively, while in *Enterococcus* spp., they are both ≤4 *μ*g/mL. Additionally, ebselen exhibited superior efficacy in eradicating MRSA during intracellular infection compared to vancomycin and linezolid at equivalent concentrations, with no apparent toxicity observed in cell culture (murine macrophage-like cells J774A.1) or in the *Caenorhabditis elegans* model [52].

Despite vancomycin's significance in treating infections caused by multidrug-resistant Gram-positive pathogens, it poses certain toxicity risks, notably nephrotoxicity [73]. Similarly, linezolid may induce various adverse effects in certain patients, such as lactic acidosis, myelosuppression, optic or peripheral neuropathies, and myopathies [74].

AbdelKhalek et al. [58] compared the in vitro activity of ebselen with that of linezolid against 27 clinical isolates of *Enterococcus*, predominantly VRE, finding similar MIC50 and MIC90 values for both drugs. Conversely, Boyd et al. [54] observed that the activity of ebselen (0.25 to 1.0 *μ*g/mL) against clinical isolates of *S. aureus* with varying antibiotic resistance patterns was comparable to or superior to that of the most active among nine antibiotics previously tested for the strains, including oxacillin, clindamycin, erythromycin, gentamicin, trimethoprim/sulfamethoxazole, doxycycline, tetracycline, vancomycin, and ciprofloxacin. Among these antibiotics, several can induce toxicity in patients, such as aminoglycosides such as gentamicin (ototoxicity and nephrotoxicity), tetracyclines (gastrointestinal disorders, inflammatory reactions such as esophagitis, pancreatitis, and ulcers, brown staining of teeth, and bone retardation in babies and children), and fluoroquinolones (nausea, vomiting and abdominal discomfort, and side effects on the central nervous system) [74].

Certain antibiotics, including clindamycin, fluoroquinolones, cephalosporins, and vancomycin, may disrupt the microbiota, potentially leading to the uncontrolled proliferation of *Clostridioides difficile* and consequent pseudomembranous colitis [75]. While the effect of ebselen on the microbiota remains underexplored, Garland et al. [76] demonstrated that oral administration of ebselen to hamsters not only reduced the recurrence of *C. difficile* infection but also enhanced the recovery of microbiome diversity following antibiotic treatment.

Traditional antibiotics typically exert their antibacterial effects through various mechanisms, including inhibition of cell wall synthesis (e.g., penicillins, cephalosporins, carbapenems, and vancomycin), inhibition of protein synthesis (e.g., macrolides, tetracyclines, and aminoglycosides), DNA interference (e.g., quinolones), alterations in membrane permeability (e.g., polymyxins), and inhibition of folic acid metabolism (sulfonamides and trimethoprim), acting primarily on specific bacterial targets. For example, beta-lactams bind to penicillin-binding proteins (PBPs), hindering transpeptidation reactions necessary for cell wall synthesis, while quinolones can bind to topoisomerases, disrupting DNA repair functions and leading to DNA breaks. Aminoglycosides bind to the 30S subunit of the ribosome, inducing misreading and premature termination of mRNA translation [77].

In contrast, ebselen's primary antibacterial mechanism involves TrxR inhibition, leading to bacterial death due to oxidizing radical accumulation, particularly in bacteria lacking a redundant antioxidant system, such as those deficient in GSH. Interestingly, recent studies have suggested that certain antibiotics can induce ROS production, thereby contributing to oxidative stress and bacterial death [78–83]. Consequently, antioxidant systems like Trx and GSH would also be important in antibiotic resistance.

Kohanski et al. [83] demonstrated that the major classes of bactericidal antibiotics (quinolones, aminoglycosides, and beta-lactams) induce the production of highly detrimental hydroxyl radicals in both Gram-negative and Gram-positive bacteria, contributing to cell death. Conversely, bacteriostatic drugs do not generate hydroxyl radicals. The authors concluded that the mechanism of hydroxyl radical formation induced by bactericidal antibiotics involves an oxidative damage cellular death pathway, including tricarboxylic acid cycle disruption, transient NADH depletion, destabilization of iron-sulfur clusters, and stimulation of the Fenton reaction. Additionally, Po et al. [84] attributed the bactericidal mechanism of daptomycin in *S. aureus* to ROS production through interactions with the bacterial cell membrane, as well as the binding of daptomycin to the Usp2 protein. By suppressing this protein's function, the organism becomes unable to express an anti-ROS response, rendering it highly susceptible to excessive ROS production. Recently, Ki et al. [85] reported that *E. coli *single-gene knockout strains with reduced ROS scavenging exhibit increased ROS accumulation and quicker resistance acquisition when exposed to sublethal levels of bactericidal antibiotics.

According to Thangamani [16], ebselen demonstrated synergistic activity with topical antibiotics (mupirocin, fusidic acid, retapamulin, and daptomycin) against various resistant strains of *S. aureus*. The degree of synergy was assessed after 12 hours of treatment with ebselen (0.0312 *μ*g/mL) in combination with subinhibitory concentrations of topical antimicrobials. The authors proposed that combining ebselen with topical antimicrobials could serve as a promising strategy for treating staphylococcal skin infections and reducing the likelihood of strains developing resistance. Similarly, ebselen was found to exhibit significant synergistic activity with all conventional antimicrobials tested in vitro against MRSA USA300 in another study [52]. However, synergistic activity with ebselen was not observed with linezolid, vancomycin, chloramphenicol, or gentamicin in eliminating intracellular MRSA.

Given the potential contribution of antibiotic-induced oxidative stress to bacterial death, it is plausible to hypothesize that this may represent a factor contributing to the synergistic activity observed in combining ebselen with traditional antibiotics.

## 5. Ebselen's Effects on Bacteria and Fungi

The success of the microbial pathogen in causing disease is closely related to the production of its virulence factors, which can mediate adhesion, invasion, aggression, and survival against the host's defense factors. Besides acting as an antimicrobial, ebselen has been shown to inhibit some microbial virulence factors. Many molecules that act on virulence are protein-based, and in addition to inactivating bacterial thioredoxin reductase, ebselen can also act by inhibiting protein synthesis, such as exotoxins.

### 5.1. Ebselen's Effect on Virulence Factors of Gram-Positive Bacteria

*Clostridioides difficile*, a Gram-positive spore-forming rod, is the main cause of antibiotic-associated nosocomial diarrhea, and its pathogenesis results from the activity of two toxins: TcdA (toxin A) and TcdB (toxin B) [86]. Ebselen has been shown to directly inhibit TcdA and TcdB by forming a covalent bond with the active site cysteine in the cysteine protease domains of these toxins. Moreover, the toxin's biosynthesis was also substantially affected by ebselen at its MIC [61]. In MRSA, ebselen significantly suppressed the production of Panton–Valentine leukocidin (PVL) and *α*-hemolysin (Hla) toxins [16].

Another important factor associated with bacterial virulence is the biofilm, which not only protects against the host's immune response but also prevents antimicrobials from reaching the pathogen. Against the VRE biofilm, ebselen demonstrated the ability not only to inhibit biofilm formation but also to disrupt the mature biofilm [58]. The efficacy of ebselen in reducing established biofilms formed by *S. aureus* and *S. epidermidis*, prominent causative agents of implant-associated hospital infections, surpassed that of conventional antibiotics including linezolid, mupirocin, vancomycin, and rifampicin [16]. However, the molecular mechanisms underlying ebselen's antibiofilm action on Gram-positive bacteria remain unknown.

### 5.2. Ebselen's Effect on Virulence Factors of Gram-Negative Bacteria

Ebselen is a potent inhibitor of bacterial ureases [87], enzymes that can play a fundamental role in the virulence of some microorganisms. For example, the survival of *H. pylori* in the stomach depends on the production of this enzyme, which converts urea into CO_2_ and ammonia, neutralizing acidity and creating a favorable pH for gastric colonization by the pathogen. Furthermore, the urease of *H. pylori* contributes to the loss of the integrity of tight junctions in the epithelium and is an inflammatory inducer [88]. In *Proteus mirabilis*, the production of urease increases the pH of the urine. It causes the precipitation of soluble polyvalent anions and cations, resulting in the formation of struvite or apatite stones [89]. In addition, urease-negative mutants of *P. mirabilis* can colonize the urinary tract, and this colonizing capacity is around 100 times lower than that in the parental strain [90]. Ebselen has been found to inactivate *H. pylori* urease with Ki in the nanomolar range [87], and biological studies on ebselen-derived compounds have shown potent inhibition of ureolysis in whole *P. mirabilis* cells in a urine model [91].

Ebselen also exhibits an impact on biofilms produced by Gram-negative bacteria, although the underlying molecular mechanisms remain incompletely understood. Utilizing the crystal violet assay in microtiter plates, Shaikh [67] demonstrated significant inhibition of biofilm formation on *Neisseria mucosa* by ebselen. Moreover, the biofilm inhibition capacity of ebselen was further affirmed through an assay indicating reduction in the hydrophobicity of the bacterial cell surface, a crucial factor in bacterial adhesion and thus inhibiting an initial step in biofilm formation. Additionally, the eradication percentage of preformed *N. mucosa* biofilms increased with escalating concentrations of ebselen. Quantification of extracellular matrix components of biofilms treated with ebselen revealed that the likely mechanism of this reduction involved the drug's capability to degrade extracellular DNA (eDNA) within the layer of extracellular polymeric substance (EPS) present in the biofilm, confirming ebselen's ability to disrupt the EPS matrix of matured biofilms. Furthermore, ebselen also attenuated the quorum sensing pathway (QS), as indicated by decreased activities of urease and protease enzymes, which are bacterial virulence factors regulated by the QS.

QS represents an intercellular communication system that regulates the production of several virulence factors and biofilm formation in numerous microbial pathogens, rendering it a significant potential target for drug intervention. Generally, the QS system involves the production and release of chemical signaling molecules known as autoinducers (AIs), which attain a certain concentration threshold as a consequence of bacterial growth. These AI molecules subsequently bind to transcriptional regulators, thereby activating or repressing specific genes. Compounds that disrupt QS pathways are termed QS inhibitors (QSIs). QSIs can act by suppressing AI synthesis, functioning as an antagonist of the regulator, or preventing the regulator from binding to DNA [92]. The specific anti-QS mechanism of ebselen is yet to be elucidated.

In another study akin to the aforementioned one, Shaikh et al. [93] demonstrated that ebselen impeded *Serratia marcescens* biofilm attachment by significantly reducing cell surface hydrophobicity. Additionally, it proved effective against preformed biofilms by degrading the eDNA component of the EPS matrix, as assessed through quantification of biofilm matrix components and crystal violet assay and validated by scanning electron microscopy analysis. Furthermore, ebselen led to decreased production of QS-controlled virulence factors, including urease activity and prodigiosin pigment production. Additionally, ebselen inhibited swimming and swarming motility of *S. marcescens*, both regulated by the QS inducer acyl homoserine lactone (AHL). Molecular docking analysis validated the strong binding of ebselen to specific QS proteins (1Joe and PigG) of *S. marcescens* via hydrogen bonds and aromatic interactions, indicating its potent antibiofilm potential [93].

In *P. aeruginosa*, a significant opportunistic pathogen, ebselen has been identified to act as an antivirulence factor by inhibiting the cyclic di-GMP (cdiGMP) signaling pathway, which regulates biofilm formation and flagellum-mediated motility. Lieberman et al. [94] introduced a rapid and quantitative high-throughput screen for inhibitors of protein-cdiGMP interactions based on the differential radial capillary action of ligand assay (DRaCALA). Through this approach, ebselen was identified as an inhibitor of cdiGMP binding to receptors containing an RxxD domain, including pellicle polysaccharide (PelD) and diguanylate cyclases (DGC). The compound and its oxide covalently modified cysteine residues, thus reducing DGC activity. Treatment of *P. aeruginosa* with ebselen and ebselen oxide reversed cdiGMP regulated phenotypes, including motility and biofilm formation [94]. Moreover, Kim et al. [95] demonstrated that ebselen, ebselen oxide, and ebsulfur inhibited alginate production by *P. aeruginosa*. Alginate, a viscous exopolysaccharide produced by strains of *P. aeruginosa*, is associated with chronic lung infections, resulting in a poor prognosis in patients with cystic fibrosis. Besides transcriptional regulation, alginate biosynthesis necessitates allosteric activation by cdiGMP binding to an Alg44 protein. Ebselen and ebsulfur can covalently modify the cysteine 98 residue of Alg44, preventing its ability to bind cdiGMP [95].

### 5.3. Ebselen's Effect on Fungal Virulence Factors

Fungi such as *Candida* and *Cryptococcus* can produce biofilm on inert and biological surfaces. As with bacteria, the fungal biofilm is a protective strategy. For example, cells from strains of *C. albicans* harbored in biofilms showed survival to fluconazole at concentrations greater than 1,024 *μ*g/mL. Meanwhile, the MIC of most planktonic counterparts ranged from 0.25 to 4 *μ*g/mL [96]. Therefore, discovering drugs with antibiofilm action for fungi is just as important as for bacteria.

Ebselen showed activity with IC50 of biofilm of *C. auris* ranging from 5.8 to 9.7 *μ*g/ml. Meanwhile, *C. albicans*, *C. dubliniensis*, *C. parapsilosis*, *C. tropicalis*, *C. glabrata*, *C. lusitaniae*, and *C. krusei* ranged from 2 to 8 *μ*g/ml [34]. For *Cryptococcus neoformans*, an important opportunistic agent of meningoencephalitis, ebselen was able to block biofilm formation at a concentration of 20 *μ*g/mL^−1^ [97]. Another very important virulence factor for *Cryptococcus* is the capsule, which protects against phagocytosis, downregulates inflammatory cytokines, and protects against reactive oxygen species [98]. In an in vitro study, Mayer et al. [97] reported that ebselen—25 *μ*M (∼6.85 *μ*g)—inhibited capsule production in *C. neoformans*.

## 6. Pharmacological Aspects and Toxicity of Ebselen for Humans

The antioxidant effect of ebselen arises from its ability to mimic glutathione peroxidases (GPx) mode of action. GPx, selenoenzymes, function to protect cellular components against damage induced by the accumulation of ROS. This protective function is executed through the catalytic reduction of peroxides, involving the consumption of glutathione (GSH). Excessive ROS formation in certain diseases can overwhelm GPx activity, and so molecules such as ebselen that mimic this selenoenzyme are of interest for restoring redox homeostasis. However, unlike GPx, ebselen has the ability to react with other thiols and thus may have additional functions beyond those of GPx [99, 100]. Therefore, ebselen serves not only as an excellent ROS scavenger but also as a multitarget compound with significant effects on inflammation, apoptosis, cell differentiation, immune regulation, and neurodegenerative diseases, in addition to possessing antimicrobial, detoxifying, and antitumor activity [101].

Data on the in vivo distribution, metabolism, and excretion of ebselen are not yet fully known. In the plasma, ebselen is bound to albumin and it can be distributed to different tissues. Methylselenobenzanilide and glucorylselenobenzanilide are the primary metabolites identified in the blood, bile, or urine of rodents and humans [12, 102]. Despite the myriad possible interactions of ebselen and its metabolites with target compounds, ebselen exhibits very low toxicity due to the lack of release of inorganic selenium in the mammalian body. This suggests that its pharmacological effects are mediated by itself or by certain intermediates containing the selenium atom in an organic form, i.e., covalently bound to carbon [24, 102].

Ebselen is readily absorbed following oral ingestion and has been deemed safe for human use in several clinical trials. Clinical studies have demonstrated the potential utility of this organoselenium as an alternative to currently available drugs for various medical conditions [17–25]. However, it has not yet received official approval for the treatment of any specific disease. In a clinical trial conducted on patients with acute ischemic stroke, including thrombosis and embolism, treatment with 300 mg/day of ebselen was suggested to protect the brain from ischemic insults. No statistical difference was noted in terms of side effects between the treated group and the placebo group [23]. In another randomized, double-blind, placebo-controlled trial conducted with patients suffering from complete occlusion of the middle cerebral artery, treatment with 300 mg/day of ebselen also indicated a neuroprotective effect from ischemic damage in acute stage. The overall incidence of adverse reaction and abnormal laboratory findings was similar in the ebselen and placebo groups [103].

A phase I clinical trial involving 32 healthy subjects demonstrated good tolerability to ebselen, even at high doses of up to 1600 mg, with no discernible trends in overall adverse effects compared to placebo. Plasma selenium concentrations showed correlation with plasma ebselen levels and only 11% of the ebselen dose was excreted in urine [22].

Based on the positive results of the phase I trial, a single-center, randomized, double-blind, placebo-controlled phase II trial was conducted to evaluate the safety and efficacy of ebselen for the prevention of noise-induced hearing loss in young adults. In this study, participants were randomly assigned to receive ebselen at doses of 200 mg, 400 mg, or 600 mg, or placebo, administered twice daily. Ebselen treatment was well tolerated at all doses, with no significant differences noted in hematological, serum chemistry, or radiological assessments between the treated and placebo groups. At a dose of 800 mg/day (400 mg, twice), ebselen treatment was deemed safe and effective in preventing noise-induced hearing loss [20].

The inhibition of inositol monophosphatase (IMPase) by ebselen has also been explored for the treatment of bipolar disorder. A randomized, double-blind, placebo-controlled, crossover design clinical study with healthy participants receiving ebselen (600 mg twice daily for 2 days) indicated reduction of myo-inositol in brain regions associated with emotional processing, consistent with IMPase inhibition. In this study, higher plasma selenium concentrations were observed in the ebselen-treated group compared to placebo, confirming the oral bioavailability of ebselen in humans. Furthermore, participants reported minimal adverse effects with ebselen, like those observed with placebo [25]. Comparable results were obtained in a short-term treatment study assessing the effects of ebselen on impulsivity and emotional processing, where a decrease in inositol levels in the anterior cingulate cortex, suggestive of IMPase inhibition, and a positive bias in emotional processing were noted. Ebselen was well tolerated by patients in this study, with the only reported side effect, aside from placebo, being drowsiness [18]. It has also been shown that ebselen can easily permeate the blood-brain barrier [25, 104].

There are few data on the concentrations achieved by ebselen in human blood. In the phase I study carried out by Lynch and Kil [22], bioavailability for ebselen varied in a range of doses between 200 and 1600 mg, with mean maximum peak plasma concentrations varying from 30.3 ng/mL to 83.4 ng/mL, at 1.5 and 2.3 hours after ingestion, respectively. However, in a murine model [105], ebselen doses of 50 mg/kg administered orally and 1 mg/kg/h by intravenous infusion produced peak plasma concentrations of up to 12 *μ*g/mL. In another study, a single oral administration of radio-labelled 14C-ebselen to rats at a dose of 50 mg/kg revealed that the radioactivity in plasma reached the Cmax of 14.78 *μ*g equiv. to ebselen/ml at 1 hr after administration [105].

## 7. Conclusions

With the rapid increase and global spread of antimicrobial resistance in recent decades, there are currently limited effective conventional therapeutic options for treating infections by MDR pathogens. The problem is exacerbated in biofilm-associated infections because, in addition to the pathogen's original multiresistance, when present, the biofilm provides a barrier that prevents antimicrobials from gaining access.

The drug industry's inability to respond adequately to this issue has made it urgent to establish alternative approaches to controlling these infections. In this sense, further research into the effects of repurposing drugs with antimicrobial and/or antibiofilm activity on pathogens is essential to enable better treatment options than those currently available.

Ebselen is a drug with many pharmacological activities, which is already in clinical trials and has demonstrated antimicrobial action, especially against Gram-positive bacterial pathogens and fungi. With a few exceptions, Gram-negative bacteria are less susceptible to ebselen because they are supported by the GSH system, which is unaffected by the drug and allows redox homeostasis maintenance. However, more recent studies have shown that combining ebselen and silver results in a synergistic antibacterial effect, which could be an important strategy for eradicating infections caused by Gram-negative MDR bacteria. Another promising way to increase the activity of ebselen against Gram-negative bacteria involves the synthesis of polar selenated compounds.

Limited data from the literature show that the mean maximum plasma concentrations achieved by ebselen in human blood [22] may be below the MIC ranges of more susceptible pathogens. However, it is possible that higher concentrations can be reached in other sites [54]. It is also important to consider the potential of this organoselenium for topical use or in intestinal decolonization of pathogens. Furthermore, some discrepancies in the MIC are noted when comparing the investigations, which may be due to aspects related to the standardization of procedures. Therefore, more in vitro and in vivo studies are required to confirm the positive expectations for the clinical use of this drug as an effective therapeutic alternative for MDR pathogens.

## Figures and Tables

**Table 1:** In vitro antibacterial effect of ebselen on Gram-positive bacteria and mycobacteria.

Microorganism		Strain	MIC (*μ*g/mL)	Reference
*E. faecium*		Clinical {27}	2^*∗*^	[58]

*S. aureus*		Clinical {5}	0.25–1	[54]

*Enterococcus* spp.		VRE	64	[46]
*S. aureus*		MRSA	64	
		MSSA	0.5	

*S. aureus*		Clinical	2.2	[55]

*Bacillus* spp.	*B. cereus*	ATCC 14579	0.9	[56]
	*B. subtilis*	ATCC 6633	0.14	
*S. aureus*		ATCC 29213	1.1	
*M. tuberculosis*		H37 Rv	10	

*M. tuberculosis*		H37 Rv	20	[15]

*S. aureus*		USA 300/400	0.25	[57]

*E. faecalis*		Clinical {39}	0.78^*∗*^	[43]
*Staphylococcus* spp.	*S. aureus*	MRSA {60}	1.56^*∗*^	
	CNS	Clinical {33}	0.78^*∗*^	

*Staphylococcus* spp.	*S. aureus*	ATCC 25923	34	[32]
	*S. simulans*	103P	55	

*Staphylococcus* spp.	*S. aureus*	MRSA {11}	0.125–0.5	[16]
		Linezolid-resistant *S. aureus* (NRS 119)	0.125	
		Mupirocin-resistant *S. aureus* (NRS 107)	0.125	
		VRSA {11}	0.125–0.5	
		MSSA (ATCC 6538)	0.125	
	*S. epidermidis*	NRS 101	0.5	

*Enterococcus* spp.	*E. faecalis*	ATCC (49533, 7080, 49532, 14506, and 51229) (VRE)	0.25–1.0	[52]
		SF 24397/24413	0.125	
		SF28073	0.625	
		HH22/MMH 594/SV587 (VRE)	0.125	
	*E. faecium*	E1162	0.25	
		E0120 (VRE)/ERV102 (VRE)	0.5	
		ATCC 6569 and ATCC 700221 (VRE)	0.5–1	
*Staphylococcus* spp.	*S. aureus*	MRSA {6}	0.125–0.25	
		MSSA {2}	0.25	
		VISA {2}	0.125–0.25	
		VRSA {4}	0.125–0.25	
*Streptococcus* spp.	*S. pyogenes*	ATCC 1234	0.5	
	*S. agalactiae*	MNZ (938, 933, and 929)	0.5	

*Enterococcus* spp.	*E. faecalis*	ATCC 51229 (VRE)	0.5	[53]
	*E. faecium*	ATCC 700221 (VRE)		
*Staphylococcus* spp.	*S. aureus*	VRSA {15}/MRSA {15}	0.125–0.5	
		MSSA {4}/VISA {3}	0.125–0.25	
	*S. epidermidis*	NRS 101	0.5	

*M. tuberculosis*		H37Rv	100^*∗∗*^	[59]
		XDR and MDR clinical isolates	50^*∗∗*^	

{ }: number of isolates; ^*∗*^MIC_90_; ^*∗∗*^*μ*M; VRSA: vancomycin-resistant *Staphylococcus aureus*; MRSA: methicillin-resistant *Staphylococcus aureus*; MSSA: methicillin-susceptible *Staphylococcus aureus*; VISA: vancomycin-intermediate *Staphylococcus aureus*; VRE: vancomycin-resistant *Enterococcus* spp.; ATCC: American Type Culture Collection.

**Table 2:** In vivo antibacterial effect of ebselen.

Microorganism	Strain	Model/inoculation	Dose/route of administration	Reference
*E. faecium*	HM-952	C57BL/6 mice/oral inoculation	10 mg/kg/oral administration	[58]
*C. difficile*	630	Swiss Webster mice/oral gavage	100 mg/kg/oral administration	[60]
*S. aureus*	Clinical	Sprague Dawley rats/skin infection	25 mg/kg/intradermal administration	[55]
*A. baumanii*	Clinical	Kunming mice urinary tract infection	25 mg/kg Ebs plus 6 mg/kg Ag^+^/intraperitoneal administration	[62]
*Y. pseudotuberculosis*	Yp III	Kunming mice gastroenteritis/intragastric gavage	20 mg/kg Ebs plus 5 mg/kg Ag^+^/intraperitoneal administration	[63]
*S. aureus*	MRSA USA300	Obese and diabetic TALLYHO/JngJ mice/infections of pressure ulcers	2%/topical administration	[57]
*S. aureus*	MRSA USA300	*Caenorhabditis elegans* AU37 (L4-stage worms)	8 *μ*g/mL/immersion in 96-well plates	[52]
*S. aureus*	MRSA USA300	BALB/c mice skin infection/intradermal inoculation	1-2%/topical administration	[16]
*E. coli*	BC1 (UPEC)	BALB/c mice cystitis/bladders infected via transurethral catheterization	5 mg/kg Ebs plus 6 mg/kg Ag^+^/intraperitoneal administration	[64]
*E. coli*	ZY-1 (MDR)	Kunming mice peritonitis/intraperitoneal injection	25 mg/kg Ebs plus 6 mg/kg Ag^+^/intraperitoneal administration	[65]
*S. aureus*	MRSA USA200	BALB/c mice peritonitis/intraperitoneal injection	30 mg/kg/intraperitoneal administration	[53]

MDR: multidrug resistant; MRSA: methicillin-resistant *Staphylococcus aureus*; UPEC: uropathogenic *E. coli*.

**Table 3:** In vitro antibacterial effect of ebselen on Gram-negative bacteria.

Microorganism	Strain	MIC (*μ*g/mL)	Reference
*E. coli*	Clinical isolate (ESBL)	64–>128	[46]
	Clinical isolates (M*β*Ls)		
*K. pneumonia*	Clinical isolate (ESBL)	>128	
	Clinical isolate (NDM-1)		
*P. aeruginosa*	Clinical isolate	>128	

*Y. pseudotuberculosis*	Yp III	2^*∗*/*∗∗*^ (plus 0.5 *μ*M Ag^+^)	[63]

*H. pylori*	NCTC11637	3.13	[15]
	Clinical isolate		

*A. baumanii*	ATCC 17978	32^*∗*^	[66]
*E. coli*	MG1655	128^*∗*^	

Enterobacteriaceae	Reference strains	12.5–50	[43]

*E. coli*	ATCC 25922	274	[32]

*S. marcescens*	MTCC 2465	14^*∗∗∗*^	[67]

*A. baumanii*	ATCC BAA1605	16	[52]
*E. coli* O157:H7	ATCC 700728	32	
*K. pneumonia*	ATCC BAA2146	64	
*P. aeruginosa*	ATCC 9721	>256	
*S.* Typhimurium	ATCC 700720	32	

^*∗*^ *μ*M; ^*∗∗*^MIC_90_; ^*∗∗∗*^MIC_50_; ATCC: American Type Culture Collection; ESBL: extended-spectrum beta-lactamases; M*β*Ls: metallo-beta-lactamases; MTCC: Microbial Type Culture Collection; NDM-1: New Delhi metallo-beta-lactamases.
